# Mitochondrial genomic characteristics and phylogenetic analysis of *Cunninghamella elegans* (Mucorales: Cunninghamellaceae)

**DOI:** 10.1080/23802359.2026.2728260

**Published:** 2026-09-08

**Authors:** Rui Xu, Xiujuan Yin, Xinyue Fan, Qingran Zhu, Liqiang Wang

**Affiliations:** College of Pharmacy, Heze University, Heze, P. R. China

**Keywords:** *Cunninghamella elegans*, mitochondrial genome, phylogenetic analysis, cunninghamellaceae

## Abstract

*Cunninghamella*, a filamentous fungal genus with important biomedical and biochemical value, lacks any fully annotated mitochondrial genome to date. Herein, we presented the first complete mitogenome of Cunninghamella elegans, a circular 41,552 bp molecule (GC 27.86%) encoding 14 conserved protein-coding genes, 2 rRNA genes, 24 tRNA genes, and 6 non-conserved ORFs. Structural comparison with related species (*Absidia glauca* and *Gongronella* sp. w5) revealed dynamic evolution in intron and repeat elements. Phylogenetics places *C. elegans* within Cunninghamellaceae, with *Gongronella* as its closest relative. This reference mitogenome will underpin future evolutionary and taxonomic investigations of this industrially and medically significant lineage.

## Introduction

The genus *Cunninghamella* (Cunninghamellaceae) represents one of the most predominant and extensively studied groups within the Mucorales. It currently comprises 47 accepted species, according to the Species Fungorum database (accessed 17 July 2026) and recent reports (Ding et al. 2025; Jiang et al. 2025; Lima et al. 2026). First described by Matruchot in 1903 (as *Cunninghamella african*a, later *C. echinulate*) (Matruchot 1903; Zheng and Chen 2001), these typically saprotrophic fungi play a crucial role in biomedicine and biochemistry. Among the well-studied *Cunninghamella* species, *Cunninghamella elegans* Lendn. 1907 is particularly noteworthy as a microbial model for bioconversion and drug metabolism studies (Asha and Vidyavathi 2009; Ibrahim et al. 2023). Additionally, *C. elegans* can produce a broad spectrum of enzymes and bioactive compounds, with significant biological, agricultural, environmental, and industrial potential (Gonçalves et al. 2021; El-Seadawy et al. 2024).

The traditional taxonomic classification of *Cunninghamella* relies primarily on morphological characteristics and specific genetic markers such as the internal transcribed spacer (ITS) region, the large subunit (LSU), and the translation elongation factor 1 alpha (TEF1α) gene (Zhang et al. 2020; Wang et al. 2022; Ding et al. 2025; Jiang et al. 2025). In recent years, fungal mitogenomes have emerged as valuable tools for investigating phylogenetic relationships and genome evolution (Li et al. 2020; Song et al. 2020; Li et al. 2022). However, studies on Cunninghamella have focused mainly on metabolism, while mitogenome structure, gene content, and phylogeny remain largely unexplored.

Although an assembled but unannotated *C. elegans* mitogenome and several congeneric sequences exist in NCBI, none include gene boundaries, tRNA predictions, or structural organization. This study fills this deficiency by providing the first fully annotated, structurally resolved mitogenome for this species, enabling robust phylogenetic and functional analyses and clearly establishing our work’s novelty.

## Materials and methods

**Figure 1.**
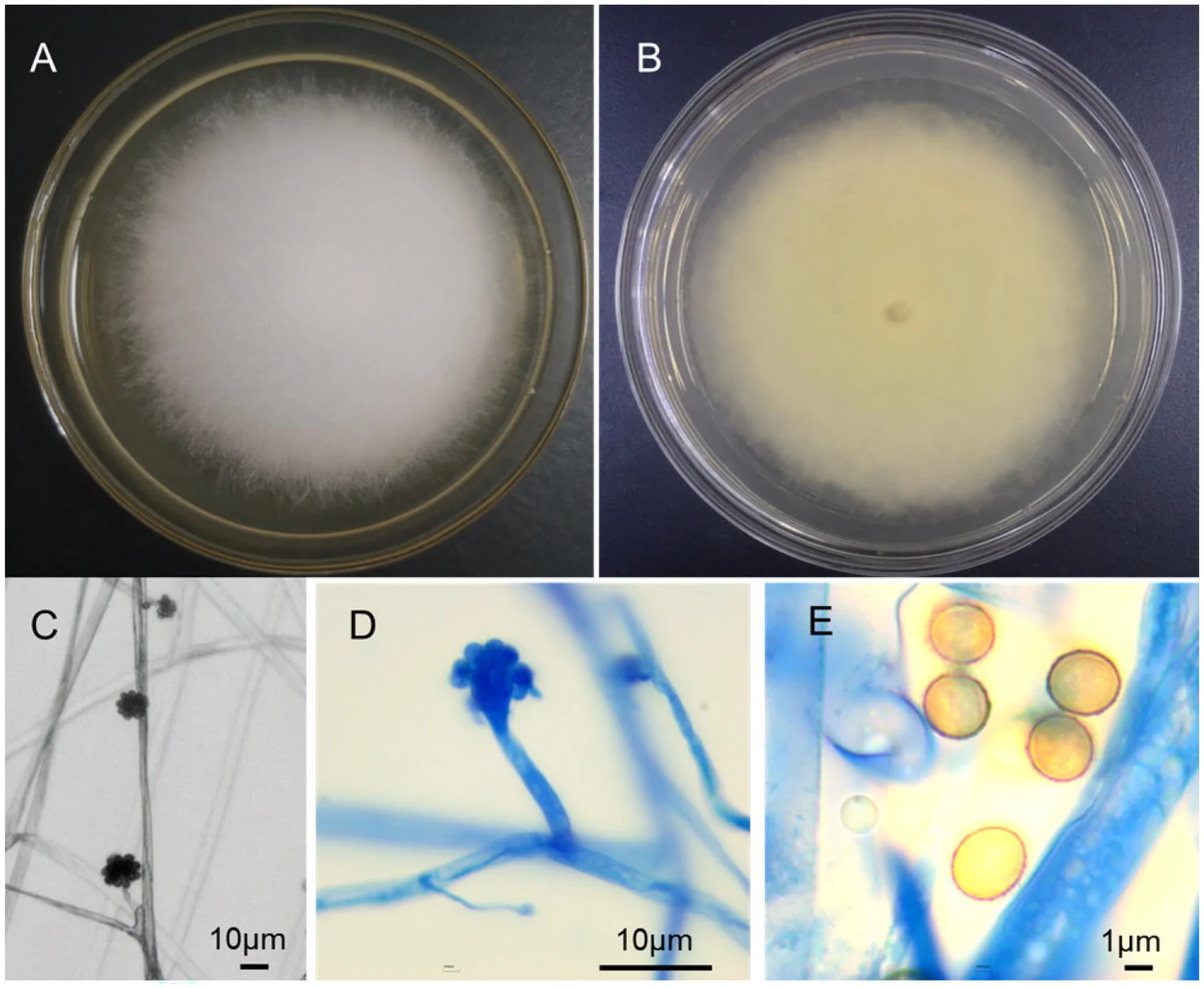
The morphologies of *C. elegans* GDMCC 3.575. (A, B) Colonies on PDA at 25 °C for 3 d, (A) obverse, (B) reverse, fast growing, initially white, gradually becoming light gray, floccose; (C) Aerial hyphae with sporangiophores and sporangia; (D) Sporangiophores and sporangia stained with lactophenol cotton blue; (E) Mature sporangiospores.

Samples of *C. elegans* strain (GDMCC 3.575) (Figure 1) were obtained from Guangdong Microbial Culture Collection Center (GDMCC) and deposited at Xurui’s lab at Heze University (N: 35°16′24″, E: 115°27′59″) under the voucher number CU001 (Rui Xu, xrxurui@yeah.net).

Genomic DNA was extracted using Fungal Genomic DNA Kit (Tiangen Biotech) from mycelium of CU001 cultures grown on PDA medium. DNA was fragmented to ∼300 bp for library construction and sequenced on DNBSEQ platform (Wuhan Benagen Technology). Raw reads were quality-checked with FastQC and filtered using Trimmomatic (Bolger et al. 2014) with default parameters. Filtered reads were assembled using NOVOPlasty (v4.3.2) with a k-mer size of 33 (Dierckxsens et al. 2017). Assembly quality and circularization were assessed *via* DrawSeqDepth, using whole-genome coverage and targeted 1,000 bp junction-flanking coverage.

The assembled genome was annotated by MFannot (Lang et al. 2023) and MITOS (Bernt et al. 2013), with the mold mitochondrial genetic code. All annotations were manually curated and refined using Apollo (Pontius 2018), and the predicted tRNA genes were further validated using tRNAscan-SE (Chan and Lowe 2019). The annotated mitogenome was subsequently submitted to GenBank under the accession number PV329827. PMGmap was employed to generate a graphical map of the mitogenome (Zhang et al. 2024).

For phylogenetic analysis, an ML tree was constructed from 14 conserved mitochondrial protein-coding genes (PCGs) of our assembly and 11 other Mucorales species (*P. verticillata* as outgroup). PCGs were aligned with MAFFT (Katoh and Standley 2016), concatenated in PhyloSuite (Zhao et al. 2025), and analyzed with IQ‑TREE (Nguyen et al. 2015) under GTR+F + I + R2 (selected by ModelFinder; Kalyaanamoorthy et al. 2017), with bootstrap support from 1,000 replicates.

## Results

**Figure 2.**
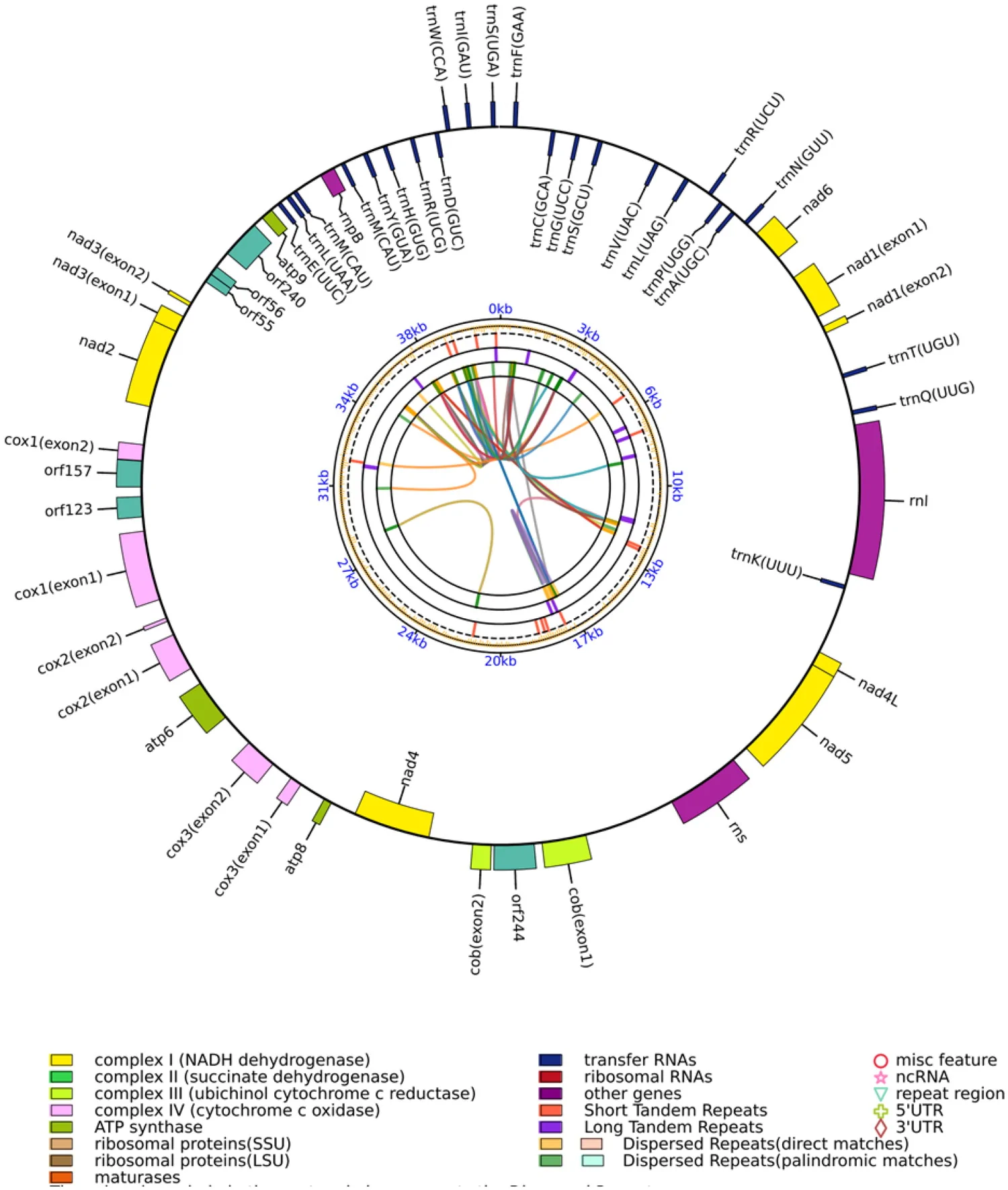
Circular map of the *C. elegans* mitochondrial genome. The mitogenome is 41,552 bp in length, with a GC content of 27.86%. genes are represented by different colored blocks. From the center outward, the first and second tracks showed the dispersed repeat sequences (direct and inverted) and their connection on the genome, marked by yellow and green arcs. The third track showed tandem repeat sequences, represented as purple bars. The fourth track displayed microsatellite repeats (SSR), represented as orange bars. The fifth track showed the GC content across the genome. The sixth track presented genes, color-coded by function (see the bottom). Outer genes were transcribed clockwise; inner genes are transcribed anticlockwise.

The whole genome DNA was successfully sequenced, yielding approximately 14.3GB of raw data (fastq format). The assembled *C. elegans* mitogenome is a circular DNA molecule with a total length of 41,552 bp (Figure 2). The average sequencing depth of the genome was 6,149.72×, as well as a minimum depth of 952× (Figure S1). The overall base composition was 35.2% A, 13.1% C, 14.7% G, and 37.0% T, with a biased GC content of 27.86%.

Analysis of the *C. elegans* mitogenome revealed a typical set of 14 conserved PCGs, including seven NADH dehydrogenase subunit genes (*nad*1-6 and *nad*4L), three cytochrome oxidase genes (*cox*1-3), three ATP synthase subunit genes (*atp*6*, atp*8, and *atp*9), and a cytochrome b gene (*cob*), along with two rRNA genes (*rnl* and *rns*), 24 tRNA genes, one RNase P RNA gene (*rnp*B) and six non-conserved open reading frames (ncORFs) (Figure 2). A total of six PCGs (*cob*, *cox*1, *cox*2, *cox*3, *nad*1, and *nad*3) were found to contain introns, with each gene harboring a single group IB or ID intron (Figure S2, Table S1). These introns ranged from 200 bp to 1,296 bp in size, and ORFs encoding LAGLIDADG or GIY-YIG homing endonucleases were identified in *cob* and *cox*1 (Table S1). In addition, three free-standing GIY-YIG ORFs were detected in intergenic regions (Table S1).

**Figure 3.**
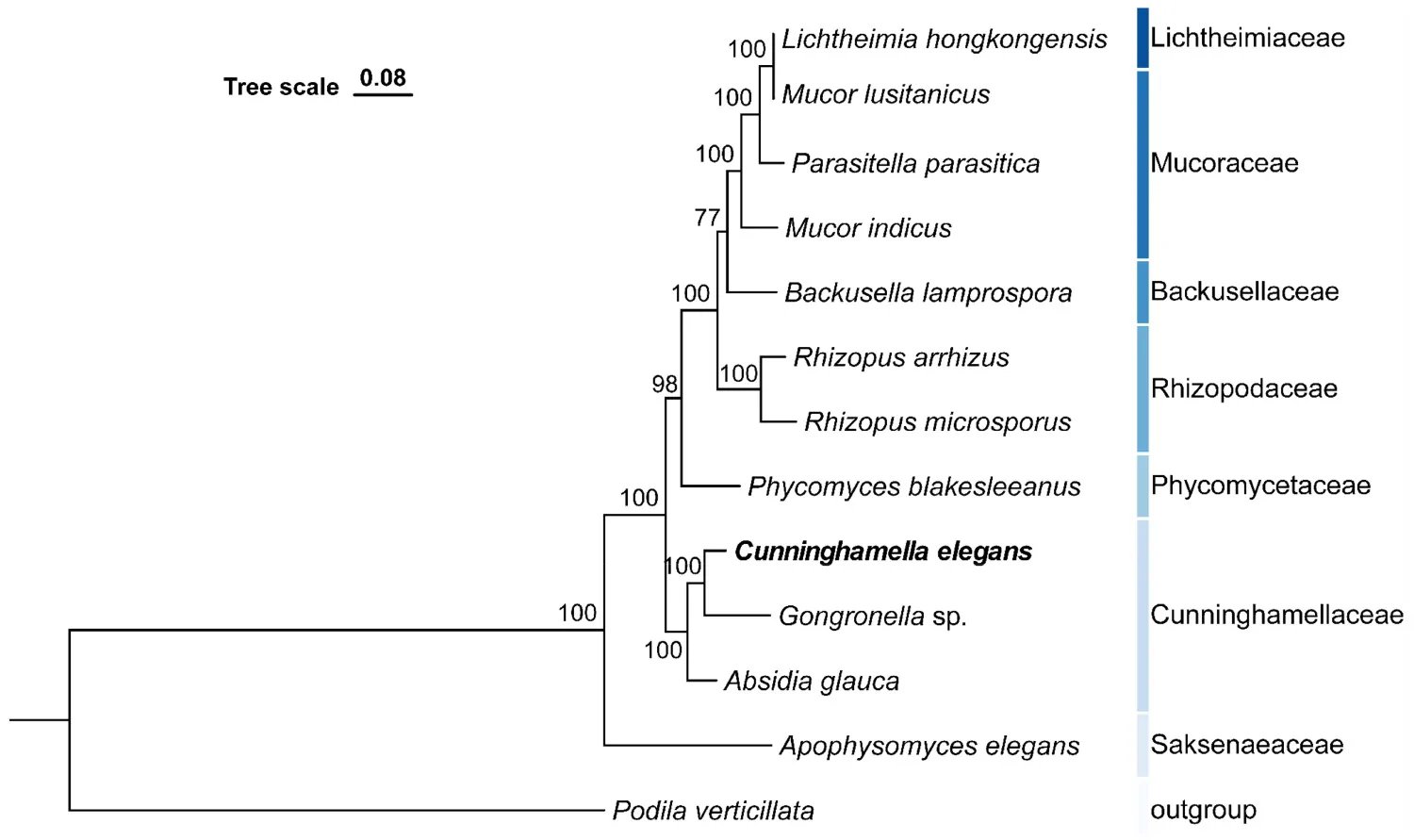
The ML phylogenetic tree of *C. elegans* and related taxa based on 14 conserved mitochondrial protein-coding genes. Bootstrap values based on 1000 replicates were shown at each node. Twelve related mucorales species and one outgroup were selected for reconstructing the ML tree. The outgroup was *podila verticillata* (AY863211). New *C. elegans* mitochondrial genome (PV329827) in this study is displayed in bold. The other 11 mucorales mitochondrial genomes were from *absidia glauca* (KU196782), *apophysomyces elegans* (MT047458), *backusella lamprospora* (OR753395), *gongronella* sp. (OK572620), *lichtheimia hongkongensis* (KJ561171), *mucor lusitanicus* (KR809877), *mucor indicus* (OR664024), *parasitella parasitica* (KM382275), *phycomyces blakesleeanus* (KR809878), *Rhizopus microsporus* (OR664025), and *Rhizopus arrhizus* (AY863212).

Since no other annotated congeneric sequences exist in GenBank, our genome is the sole *Cunninghamella* representative in the ML analysis of 14 core PCGs from 12 Mucorales mitogenomes. The resulting ML tree placed *C. elegans* in a well-supported clade with *Absidia glauca* and *Gongronella* sp. w5 (Figure 3), consistent with its classification within Cunninghamellaceae.

## Discussion and conclusion

We reported the first mitogenome of *C. elegans*, a circular DNA molecule with a compact architecture of 14 core PCGs, 2 rRNA genes, 24 tRNA genes, 1 *rnp*B gene, and 6 ORFs. Among all available *Cunninghamella* mitogenomes, our annotated assembly (41,552 bp, 27.86% GC) is slightly larger than an unannotated *C. elegans* genome (BK072837, 41,506 bp, 27.84% GC). This difference likely stems from precise boundary definition of repeat-rich regions during manual curation. Both *C. elegans* mitogenomes are larger than other congeners (34,875–39,956 bp) but fall within their GC range (25.73–28.41%) (Table S2). Inter-generic comparisons with *Absidia glauca* and *Gongronella* sp. w5 (Ellenberger et al. 2018; Zhang et al. 2022) showed that all the three species share 14 PCGs but differ structurally: *A. glauca* has the largest genome (63,080 bp), the most introns, and the most abundant LSRs, whereas *C. elegans* and *Gongronella* sp. w5 have fewer, suggesting lineage-specific expansion of these elements within Cunninghamellaceae (Table S2). Gene order was generally conserved, with lineage-specific rearrangements distinguishing each species (Figure S3).

In fungal mitogenomes, intron gain/loss is often mediated by horizontal transfer or ectopic recombination, with homing endonuclease genes promoting intron mobility (Férandon et al. 2010). The abundant LSRs and expanded introns in *A. glauca* suggest repetitive-element recombination may have driven genome expansion, while the compact genomes of *C. elegans* and *Gongronella* sp. w5 point to stronger contraction forces. Despite structural variation, all mitogenomes share 14 conserved PCGs. This reflects purifying selection on core respiratory functions. It also reinforces that fungal mitogenomes consist of a conserved core gene set. Variable accessory elements are present alongside this core (Sandor et al. 2018).

Species identification in Cunninghamellaceae is challenging due to morphological convergence and difficult nuclear gene amplification (Zhao et al. 2021; Ding et al. 2025). Our phylogenetic analysis, based on shared mitochondrial PCGs, robustly places *C. elegans* within Cunninghamellaceae. However, few annotated mitogenomes, especially in *Cunninghamella*, hamper deeper evolutionary inference. More mitochondrial data are needed to resolve higher-level relationships and genomic variation in these early-diverging fungi. As the first characterized *Cunninghamella* mitogenome, our assembly reveals conserved core genes but dynamic structures, offering a foundation for future taxonomic and evolutionary studies in this family.

## Supplementary Material

Supplemental Material

## Data Availability

The complete mitogenome sequence of *C. elegans* reported in this study was submitted to the NCBI database under accession no. PV329827. The associated BioProject, BioSample, and SRA numbers are PRJNA1235460, SAMN54501360, and SRR32695676, respectively.
