# Supplementary material for: Mitochondrial genomic characteristics and phylogenetic analysis of Cunninghamella elegans (Mucorales: Cunninghamellaceae)
Source: Mitochondrial DNA B Resour. 2026 Sep 8;11(10):1140–5. doi: 10.1080/23802359.2026.2728260 (PMC13560420; doi:10.1080/23802359.2026.2728260)
Supplement: Supplementary file 1 [file TMDN_A_2728260_SM1766.pdf]

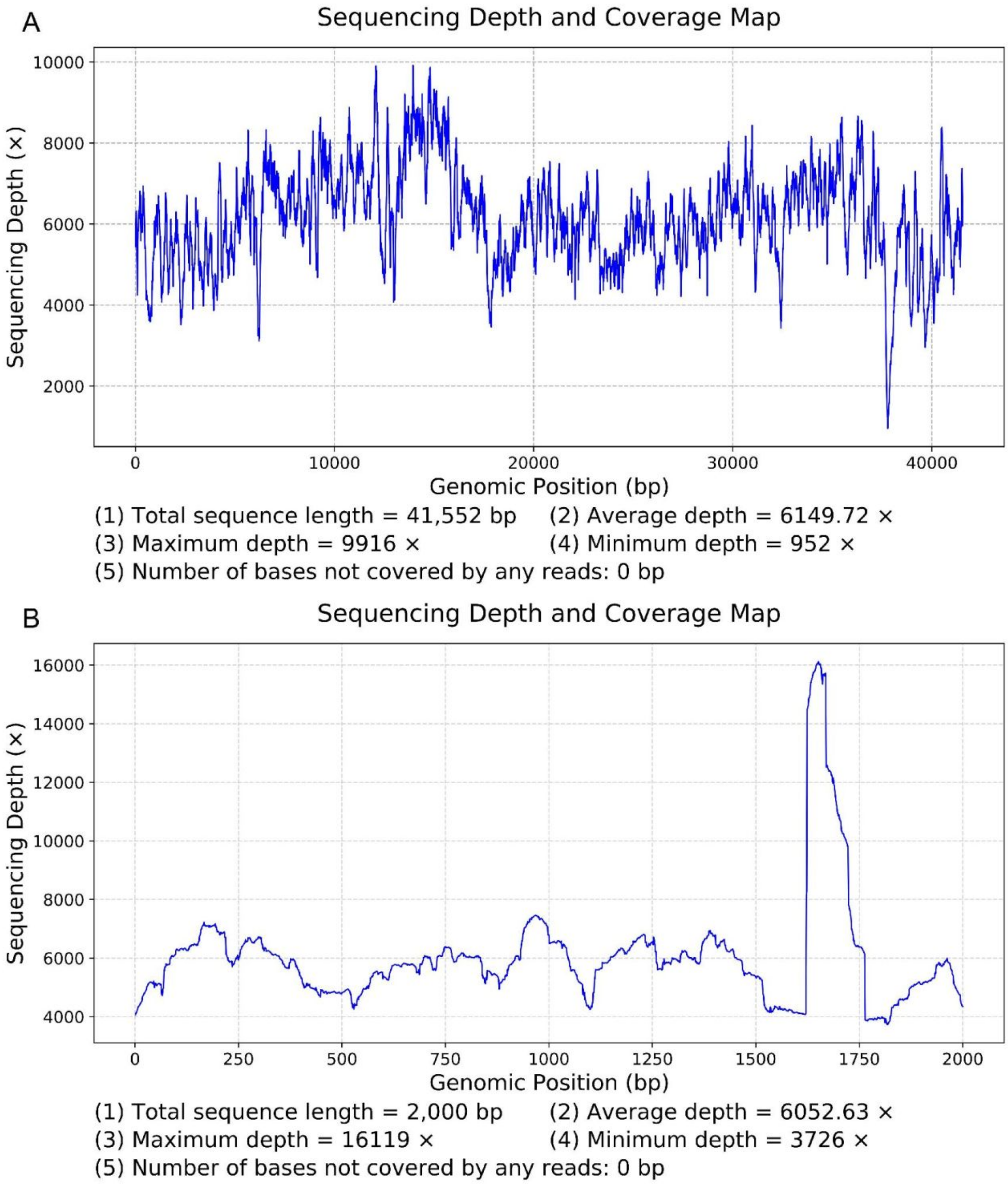

Figure S1. Sequencing depth and coverage maps validated the high-quality assembly of the *C. elegans* mitogenome. (A) Sequencing depth and coverage map of the complete *C. elegans* mitogenome; (B) Sequencing depth and coverage map across the start-end junction of the circular mitogenome. The plot displays the sequencing coverage across the *C. elegans* mitogenome. The horizontal axis represents genomic coordinates (bp position), while the vertical axis indicates read depth (coverage). The sequencing depth plot was calculated by DrawSeqDepth (https://github.com/wlqg1983/DrawSeqDepth) with default parameters.

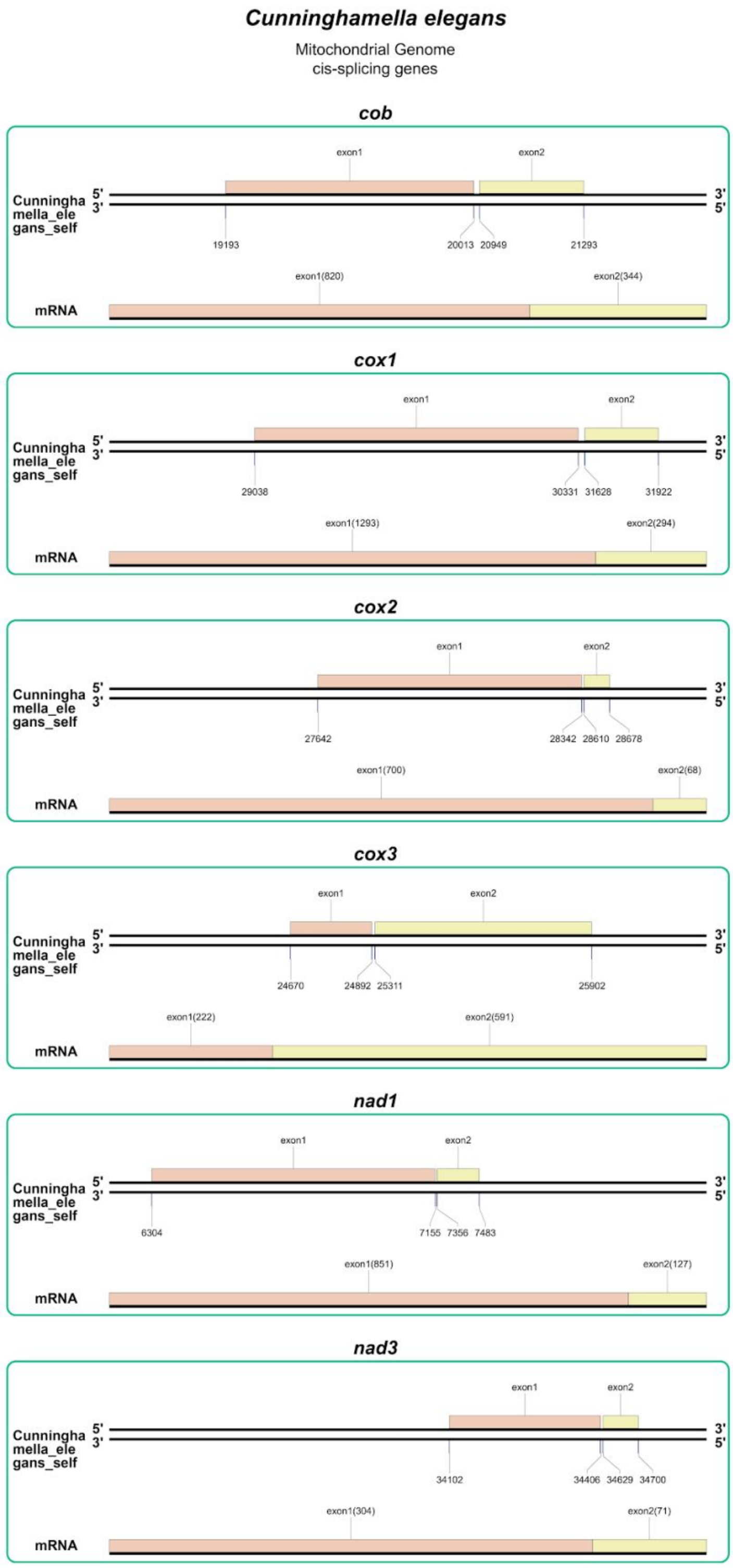

Figure S2. Gene structures of six cis-spliced intron-containing genes in the *C. elegans* mitogenome. The exon-intron organization of six mitochondrial genes with cis-spliced intron-containing (*nad*1, *nad*3, *cob*, *cox*1, *cox*2, and *cox*3) introns was visualized using the online tool PMGmap (http://47.96.249.172:16086/home/).

Table S1. Characteristics of introns and homing endonucleases in the *Cunninghamella elegans* mitochondrial genome.

Host Gene	Group	Intron Location (nt)	Size (bp)	ORF Name	Domain/HEG Family	ORF Location (nt)	Best BLAST hit	Putative function
cob	IB	20013…20947	935	orf244	LAGLIDADG	20158…20893	LAGLIDADG endonuclease [*Absidia glauca*]	Homing endonuclease; intron mobility
*cox*1	ID	30331…31626	1296	orf123	GIY-YIG	30593…30965	GIY-YIG endonuclease [*Parasitella parasitica*]	Homing endonuclease; DNA cleavage
				orf157	GIY-YIG	31144…31618	GIY-YIG endonuclease, [*Rhizopus microsporus*]	Homing endonuclease; DNA cleavage
*cox*2	IB	28342…28608	267	-	-	-	-	-
*cox*3	IB	24892…25309	418	-	-	-	-	-
*nad*1	IB	7155…7354	200	-	-	-	-	-
*nad*3	IB	34406…34627	222	-	-	-	-	-
intergenic	-	-	-	orf240	GIY-YIG	35825…36548*	GIY-YIG endonuclease [*Parasitella parasitica*]	Homing endonuclease; DNA cleavage
	-	-	-	orf55	GIY-YIG	35337…35170*	GIY-YIG endonuclease [*Absidia glauca*]	Homing endonuclease; DNA cleavage
	-	-	-	orf56	GIY-YIG	35340…35511*	GIY-YIG endonuclease [*Absidia glauca*]	Homing endonuclease; DNA cleavage

* Asterisks indicate features on the complementary strand.

For Peer Review Only

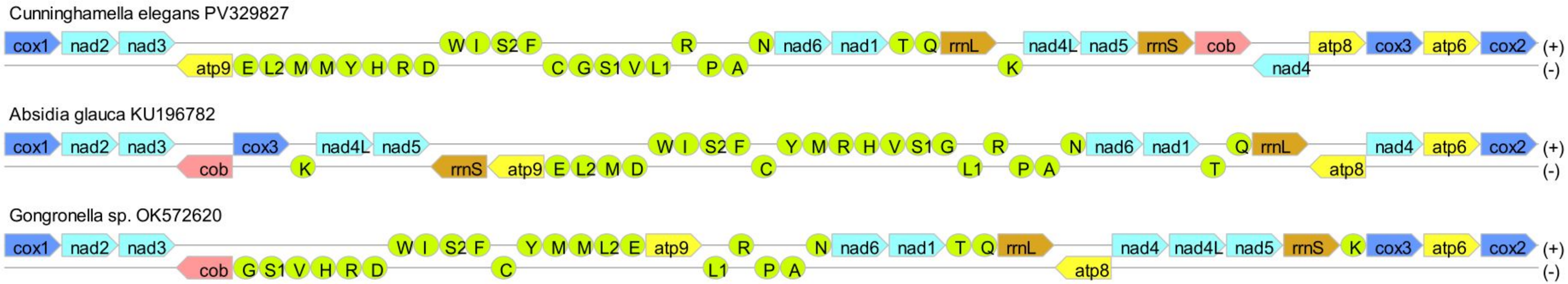

Figure S3. Mitochondrial gene order and rearrangements among three species of the family Cunninghamellaceae. The comparative gene order map was generated using the mitogenome module of PhyloSuite (v2.0) , with *cox*1 as the starting reference point. Gene categories are distinguished by different color blocks, with each gene name annotated directly on its corresponding block. The orientation of transcription is indicated by (+) and (−) signs, which correspond to the heavy (positive) and light (negative) strands, respectively.

Peer Review Only

Table S2. Comparison of mitochondrial genomic characteristics among three Cunninghamellaceae fungi, showing size, GC%, gene number, and repeat elements.

	Nucleotide Length (bp)	GC content (%)	Number of Genes					Number of introns	Repeat elements	
			Tota l	Protein	tRNA	rRNA	ORF		SSR	LSR
C. elegans (this study)	41,552	27.86	47	14	24	2	6	6	15	52
C. elegans (unannotated)	41,506	27.84	-	-	-	-	-	-	-	-
Other *Cunninghamella* spp.	34,875–39,956	25.73–28.41	-	-	-	-	-	-	-	-
Absidia glauca	63,080	28.18	54	14	24	2	14	11	3	224
Gongronella sp.	36,593	30.06	46	14	24	2	6	3	4	15

For Peer Review Only
